# Simultaneous determination of the potent anti-tuberculosis regimen—Pyrazinamide, ethambutol, protionamide, clofazimine in beagle dog plasma using LC–MS/MS method coupled with 96-well format plate

**DOI:** 10.1016/j.jpba.2019.02.006

**Published:** 2019-05-10

**Authors:** Shengyuan Wu, Liai Lan, Jingjing Jiang, Xianting Ding, Chih-Ming Ho, Yuefen Lou, Guorong Fan

**Affiliations:** a School of Medicine, Tongji University, No. 1239 Siping Road, Shanghai 200092, PR China; b Department of Pharmaceutical Analysis, School of Pharmacy, Second Military Medical University, No. 325 Guohe Road, Shanghai 200433, PR China; c Shanghai Key Laboratory for Pharmaceutical Metabolite Research, School of Pharmacy, Second Military Medical University, No. 325 Guohe Road, Shanghai 200433, PR China; d Department of Pharmacy, Shanghai Fourth People’s Hospital, Tongji University, No. 1878 North Sichuan Road, Shanghai 200081, PR China; e Med-X Research Institute, School of Biomedical Engineering, Shanghai Jiaotong University, Shanghai 200030, PR China; f Department of Bioengineering, University of California, Los Angeles, CA 90095, United States; g Department of Clinical Pharmacy, Shanghai General Hospital, Shanghai Jiaotong University, No. 100 Haining Road, Shanghai 200025, PR China

**Keywords:** LC–MS/MS, liquid chromatography tandem mass spectrometry, FSC, Feedback System Control, PRS, Parabolic Response Surface, PZA, pyrazinamide, EMB, ethambutol, PTO, protionamide, CFZ, clofazimine, MRM, multiple-reaction monitoring, ESI, electrospray ionization, TB, tuberculosis, AIDS, acquired immune deficiency syndrome, HIV, human immunodeficiency virus, Anti-tuberculosis drug, Pharmacokinetics, Beagle dog plasma, LC–MS/MS, 96-well format plate

## Abstract

Tuberculosis is one of the top concerns in the world and acutely threatens human health. A new potent candidate regimen containing pyrazinamide (PZA), ethambutol (EMB), protionamide (PTO) and clofazimine (CFZ) was proposed by Parabolic Response Surface/Feedback System Control (FSC/PRS) system and showed excellent outcomes in vitro and vivo studies. Here, a convenient liquid chromatography coupled with tandem mass spectrometry (LC–MS/MS) method was developed for the simultaneously determination of four compounds in beagle dog plasma. The plasma samples, 50 μL for each, were pretreated by methanol on 96-well format plates and a further dilution step was designed to reduce predictable matrix effect and lessen the burden of subsequent analysis. The chromatographic separation was achieved on an Agilent SB-Aq column (4.6 mm × 150 mm, 5 μm) at 30 °C by a gradient elution within 6 min. The mobile phase was a mixture of 0.2% formic acid-5 mM ammonium acetate aqueous solution (phase A) and 0.2% formic acid methanol (phase B) with a total flow rate of 1 mL/min. The 30% of post-column eluant was injected into mass spectrometer, equipped with electrospray ionization (ESI) source under positive mode and multiple-reaction monitoring (MRM). This quantification method was proved to be satisfied in selectivity, accuracy, precision, linearity (r^2^ > 0.998), recovery, matrix effect and stability. Under the specialized conditions, the calibration curves ranged from 20 to 5000 ng/mL for PZA, 1 to 500 ng/mL for EMB, 1 to 500 ng/mL for PTO, and 1 to 200 ng/mL for CFZ. The quantitative accuracy was further assessed under different degrees of hemolyses in detail. This method was proved to be robust and efficient, and successfully applied to the pharmacokinetic study of the new regimen in Beagle dogs.

## Highlights

• LC–MS/MS method for determination of Pyrazinamide, Ethambutol, Protionamide and Clofazimine in Beagle Dog Plasma.
• Method validation was conducted according to FDA and NMPA guidelines.
• Hemolysis effect was investigated in detail.
• The method is robust and high throughput cooperated with 96-well format plates.

## 1 Introduction

The tuberculosis (TB) is among the top list of global infectious disease and *Mycobacterium tuberculosis* (Mtb) is the pathogenic bacterium of TB [1]. In 2017, an estimated 10 million new TB cases arose worldwide, and 1.6 million died [1]. TB ranks the first among all the killers in AIDS/HIV victims and approximately 35% of HIV deaths were due to TB in 2017 [1]. A multi-drug regimen according to the Mtb susceptibility is the routine therapy for TB patients with a prolonged course more than 1 month. The accompanying side effects of multiple organs, especially for liver and gastrointestinal tract, seriously interrupt the treatment, and the discontinuous course and poor patient compliance are the key factors for Mtb resistance and relapse. The multidrug-resistant TB (MDR-TB) is another severe challenge and a sustained threat, with more than half a million new MDR-TB cases were confirmed in 2017 and had to receive non-first-line therapy with less effective [1,2]. Hence, more potent chemical entities and regimens are urgently needed to conduct a shorter course of treatment for both sensitive and MDR-TB to promote clinical efficacy and lessen side-effect burden [3]. Bedaquiline (approved by U.S. Food and Drug Administration) and Delamanid (approved by European Medicines Agency) are only two new compounds approved to counter the TB. However, several safety concerns are persistently alarming like drug interactions on CYP3A4, drug related hepatic disorders, and especially cardiovascular risks and deaths, and the clinical application is partly restricted in combination [4]. Developing a brand-new compound for TB is a costly and endless course, generally containing preclinical study, phase I, II and III. In view of the multidrug treatment has been widely accepted and recommended by World Health Organization (WHO), it may be a valuable approach to regroup the marketed and under-developed drugs to exploit the latent synergistic effect and maximize anti-TB efficacy. Strictly speaking, the current multidrug regimen is proposed on the basis of empiric evidence without detailed studies of optimal combinations or dose proportions. But to identify the most efficacious 3-/4-drug combinations under optimal drug-dosage ratios, huge and tedious tasks are required because of a big candidate pool of anti-TB drugs (marketed and under developed) and the number of combinations can be exponentially large.

Recently, Ho’s et al. have developed an algorithm, called Feedback System Control (SFC) or Parabolic Response Surface (PRS), to search the optimal drug-dose combination from a large candidate drug pool [[5], [6], [7], [8], [9], [10], [11], [12]]. Based on the second order algebraic equations and experimental date, the special algorithm can plot out the drug-dose-efficacy landscape and rapidly home in on optimal solution without burdensome process [11,12]. And this outstanding platform has been used to identify optimal drug regimens in several biological systems, including antiretroviral therapy for HSV and cancer chemotherapy [[5], [6], [7], [8], [9], [10]]. For TB therapy, a new combination consisting of pyrazinamide (PZA), ethambutol (EMB), protionamide (PTO) and clofazimine (CFZ) was selected and proposed by this method and the new one shows exceeding bactericidal or bacteriostatic ability in vitro and is worth for furthermore development [11,12].

PZA, one of the first-line drugs, is fundamental for clinical standard regimens with the most excellent sterilizing ability to latent Mtb, and the only killer in acid environment [[13], [14], [15]]. Since its introduction to the treatment, the therapeutic course has been shortened to six months [15]. EMB is another first-line drug administrated during initial period. Other than PZA, the target of EMB is the biosynthesis of arabinogalactan, damaging the integrity of Mtb cytoderm [16]. PTO belongs to thioamide homologous series and serves as the core option of second-line anti-TB compounds for MDR-TB cases with the resistance to front-line therapeutics like isoniazid or rifampin [1]. CFZ is also one of the typical non-first-line drugs for MDR-TB and the good safety with no serve toxicity is attested by the long therapeutic history of leprosy, although the reversible skin discoloration happens frequently [1]. Regardless the mechanisms and physiological targets have not been clarified, several in vitro and vivo trails have demonstrated that CFZ has good efficacy against MDR-TB in different models, making CFZ an intriguing candidate for the management of drug-resistant patients [17]. Some researches have uncovered the synergism activity among CFZ and other anti-TB drugs, especially co-administrated with EMB and PZA [19,20]. The treatment guidelines for drug-resistant TB of WHO in 2016 highlighted the capacity of CFZ and the CFZ-contained regimen (including PZA, EMB, PTO, CFZ, kanamycin acid and isoniazid) for MDR-TB patients for the shorter treatment course, low cost and accessibility [18]. One most effective regimen containing these four compounds for MDR-TB patients with a minimum period of nine-month treatment has been validated for naive MDR-TB patients by a clinical trial in Bangladesh from 1997 to 2007 [21]. And this treatment has since been adopted by many low-income countries, including Cameroon [22] and Niger [23]. All these clinical studies have demonstrated impressive treatment outcomes, with success rates ranging from 84% to 89% [24]. Furthermore, these drugs are ready-made on the formulary and easy access to in the hardest-hit areas. Compared with the routine therapy for tuberculosis patients, this new regimen proposed by Ho’s group showed the excellent performance in sterilizing effect in vitro and vivo [11,12]. Hence, the combination of PZA, EMB, PTO and CFZ maybe a new chance for the whole world to counter the tuberculosis. However, according to our surveys, no report uncovered the PK properties of these four drugs in combined therapy.

To date, numerous analytical methods have been used for anti-TB drugs determination alone or in combination. Yet, the established methods suffer in some cases from the limited quantitative range, tedious sample preparation, huge sample volume, which make them less suitable for routine application [[25], [26], [27], [28]]. However, in consideration of the huge dispersion in physicochemical property of the compounds in this new regimen, it is also a big challenge to obtain suitable chromatographic behaviors on a single chromatographic column with tiny bio-sample volume during one LC or LC–MS/MS loop. This is the first time to propose PZA-EMB-PTO-CFZ combination therapy and none of the methods allows their simultaneous analysis in biological matrix within a single loop. So, it is therefore imperative to develop a robust and rapid method for this vacancy.

In this assay, a high-throughput and robust LC–MS/MS method coupled with 96-well protein precipitation plates for simultaneous determination of the PZA-EMB-PTO-CFZ combination was established and fully verified. This method has been successfully applied to the study of the pharmacokinetics of the new anti-TB regimen in Beagle dogs.

## 2 Materials and methods

### 2.1 Chemical and reagents

Ethambutol hydrochloride, pyrazinamide, protionamide, clofazimine and midazolam were purchased from Nation Institutes for Food and Drug Control. Methanol with liquid chromatography grade was purchased from Merck (Darmstadt, Germany). Ammonium acetate and formic acid were acquired from ANPEL Lab Tech. (Shanghai, China). The pure water (18.2 MΩ/cm) in this assay was deionized by a Milli-Q System (Millipore, Bedford, MA, USA). All solutions were ultrasonically degassed before use.

### 2.2 Liquid chromatographic and mass spectrometric conditions

Samples were analyzed by an HPLC-MS/MS system composed of a Shimadzu 20A solvent management system and an AB SCIEX API 4000 mass spectrometer.

The chromatographic separation was performed on an Agilent SB-Aq column (150 mm × 4.6 mm, 5 μm i.d., USA) with a total run time of 6 min. The mobile phase was composed of water phase (water-formic acid (100:0.2, v/v)-ammonium acetate 5 mM) and organic phase (methanol-formic acid (100:0.2, v/v)). The gradient profile used started with 10% B for 0.5 min, followed by a linear gradient ascent to 35% B at 0.6 min, maintained until 1.0 min and a second gradient ascent to 40% B in 0.5 min. Next a third gradient ascent to 80% B at 1.6 min and held constant over 3 min, and finally returned back to initial condition of 10% B at 4.6 min followed by a 1.5-min re-equilibration. The injection volume of each sample was 10 μL and chromatographic separation was performed at 30 °C. And the total flow rate was maintained at 1 ml/min

In mass spectrometer, four compounds and IS were ionized through electrospray ionization source under positive mode with following source parameters: 5500 V for ionizing voltage, 500 °C for source temperature, 20 psi for curtain gas and 20 psi for nebulizer gas. The compound-dependent parameters of five MRM channels, including declustering potential (DP), entrance potential (EP), collision energy (CE) and cell exit potential (CXP), were optimized individually to acquire maximal signal and list respectively in Table 1. The dwell time for each MRM channel was set at 100 ms.

**Table 1:** Summary of MS/MS parameters: precursor ions, fragment ions, declustering potential (DP), entrance potential (EP), collision energy (CE) and cell exit potential (CXP) for analytes and IS.

Compound	Transition (*m*/*z*)	DP (V)	EP (V)	CE (V)	CXP (V)
Pyrazinamide	124.1 → 79.0	85	9	28	10
Ethambutol	205.1 → 116.1	60	5	20	20
Protionamide	181.0 → 121.1	55	6	23	13
Clofazimine	473.8 → 431.4	120	5	48	30
Midazolam (IS)	326.1 → 291.1	120	9	36	20

### 2.3 Stock solution, calibration standard and quality control samples in dog plasma

Methanol was chosen for the only solvent in view of the unsatisfied solubility of EMB in acetonitrile. The stock solutions of PZA, EMB, PTO and midazolam (IS) at 1 mg/mL for each were prepared in methanol: water (8:2, v/v). The stock solution of CFZ was prepared in methanol at 0.5 mg/mL. Before sample preparation, all solutions of analytes, except for IS solution, were diluted sequentially with methanol: water (8:2, v/v) to obtain hybrid working standard solutions for calibration standards and quality control samples (QCs). Internal standard working solution (midazolam, 500 ng/mL) for samples processing was prepared in methanol. All the stocks solution and intermediate working solution were stored at −20 °C and warmed to room temperate before use.

96-well Sirocco Protein Precipitation plates (Waters corp., Milford, MA, USA) were applied to sample preparation for the rapid and synchronous processing. Calibration standards and QC samples were prepared by mixing 5 μL of hybrid working standard solution and 95 μL of Beagle dog plasma in 96-well plates with individual standard curve ranges as 20–5000 ng/mL for PZA, 1–500 ng/mL for EMB, 1–500 ng/mL for PTO, and 1–200 ng/mL for CFZ. The lower limit of quantitation (LLOQ) was selected according to our prediction of pharmacokinetic profile in Beagle dogs, as well as the signal to noise ratios, which should be greater than 10 to guarantee the quantitative accuracy of low-concentration samples. The upper limit of quantitation (ULOQ) set here based on the linearity response ranges of mass spectrometry and the suitable carry-over under the uttermost. QC samples were set at four different concentrations (20, 60, 450 and 4000 ng/mL for PZA; 1, 3, 40 and 400 ng/mL for EMB; 1, 3, 40 and 400 ng/mL for PTO; 1, 3, 15 and 160 ng/mL for CFZ) viz., LLOQ, low quality control (LQC), middle quality control (MQC) and high quality control (HQC).

### 2.4 Sample preparation

Sample preparation was conducted with protein precipitation (PPT) on 96-well plates to fit batch processing and a VIAFLO 96/384-channel pipettor (INTEGRA, Switzerland) was applied to liquid transfer steps. 50 μL plasma sample (calibration standards, QCs or test samples) and 150 μL methanol containing 500 ng/mL midazolam served as internal standard were transferred into 96-well plates in sequence. After plates capped, the liquid samples were mixed fiercely by a vibrational rotator for 5 min to ensure maximum efficiency of PPT and the sediment was abandoned by pressure filter. The processed filtrate was collected by another 96-well plate. To minimize the predictable serve matrix effect, 50 μL aliquots of the supernatants were subsequently diluted by 100 μL of diluent (methanol: water: formic acid (20:80:0.2, v/v/v)). After two-minute mixing and five-minute 13,800 *g* centrifugation, the sample plate was loaded into the auto-sampler waiting for analysis.

### 2.5 Validation of the method

The performance of the LC–MS/MS method was fully validated in accordance to the guidelines published by FDA and NMPA [29,30], including selectivity, sensitivity, calibration curve, accuracy, precision, recovery, matrix effect, hemolysis, carry-over, crosstalk, dilution integrity and stability.

#### 2.5.1 Selectivity

Although LC–MS/MS method possesses excellent anti-interference ability owe to MRM mode, the unpredictable interference signal does exist in some cases, such as the endogenous substrates that share the same MRM channel and improper mobile phase chosen or elution gradient designed. Hence, selectivity tests in this assay were investigated by deionized water samples (exogenous tests) and blank plasma samples (endogenous tests). For exogenous tests, six repeating analysis loops of deionized water samples were conducted continuously to reflect the chromatographic stability and whether any ghost peaks appear at the retention times of each compound. The endogenous selectivity tests were carried out by comparing the spectrogram between six blank plasma samples from different dogs and LLOQ samples to evaluate that whether any interference exist. If any, the peak area should be less than 20% of LLOQ peak area.

#### 2.5.2 Sensitivity and calibration curve

The sensitivity was measured by the signal-to-noise ratio (S/N) for each compound in biological matrix. In view of the low molecular weight of PZA, EMB and PTO, the S/N ratio should be greater than 10 for the LLOQ to guarantee the accuracy lest the baseline fluctuation disturb peak integration.

Four linearity ranges for quantitation contained eight points based on internal standard calibration. The concentration ranges were as mentioned above. In view of quite low concentration at the lower curve part, 1/x^2^ was chosen as the weighting factor and the degree of fitness described by correlation coefficient (r^2^) had to be greater than 0.995. The criterion was that the deviation of each back calculated concentrations had to be within ±15% of nominal value except for LLOQ, which had to be within ±20%.

#### 2.5.3 Accuracy and precision

Four different concentrations (LLOQ, LQC, MQC, HQC) generally covering the whole scope of standard curves were selected to perform this part of validation. Eighteen repetitive samples for each concentration were analyzed in three separate days, i.e., six repetitive samples for one day, to confirm the intra-day and inter-day accuracy and precision. The evaluation indexes for accuracy were the concentration ratios of the value calibrated by curves to the nominal value and for precision were the relative standard deviation (R.S.D.) of calculated value. The criteria for accuracy was the same as that of calibration curve, ±15% deviation for LQC, MQC and HQC and ±20% for LLOQ, and for precision (R.S.D.) was below 15% (LQC, MQC, HQC) or 20% (LLOQ).

#### 2.5.4 Recovery and matrix effect

The recovery of all analytes and IS were evaluated by the ratios of the QC peak area (LQC, MQC and HQC, n = 6) to that of matrix effect samples with equal concentration. The matrix effect samples were the post-spiked samples by adding the analytes and IS with specified concentrations to the blank matrix which had been deproteinated in advance. The recovery would be satisfied if the ratios were proved to be stable without large deviations among three concentrations.

The matrix effect tests aimed at assessing the influence of co-eluted endogenous substrate to compound MS responses, causing ion suppression or enhancement. The evaluation was carried out by the area ratios between matrix effect samples and neat standard samples at three equivalent concentrations (LQC, MQC and HQC, n = 6). Six lots of plasma from different individual were fully investigated and the inter-subject variability of matrix effect at each QC level should be less than 15%.

In return, the favorable recovery and matrix effect would guarantee and be a side proof of a good calibration curve.

#### 2.5.5 Hemolysis evaluation

A hemolysis assessment was conducted to evaluate the influence on the accuracy of quantitation. The whole blood samples were totally hemolyzed after two freeze-thaw cycles and three-minute violent vortex. After ten-minute 13,800 *g* centrifugation, the full hemolysis plasma was obtained. 1,2,5,10,20 and 50 μL hemolyzed plasma were spiked into no-hemolytic plasma to prepare a series of hemolyzed plasma samples with different extents at 0.1%, 0.2%, 0.5%, 1%, 2% and 5%. The accuracy of quantitation was evaluated at two concentrations (LQC and HQC, n = 6). QC samples in hemolyzed plasma with various degrees were treated as above mentioned. The criteria for acceptability of the accuracy data was within ± 15% standard deviation (S.D.) from the nominal values.

#### 2.5.6 Carry-over and crosstalk

Considering the large spans of quantitative curves and quite low concentrations of LLOQ, it was possible that the residues of analytes would remain in LC system. To get rid of the carry-over, the LC syringe was rinsed by methanol before and after sample injection. And the carry-over was evaluated by the response of the first zero sample just after ULOQ exactly and if peaks appeared, the area should be less than 20% of the area of a LLOQ sample. To test the carry-over further, no peak could be spotted at the retention times of four drugs in third zero sample after ULOQ.

Crosstalk tests were performed separately for each compound. The concentrations of ULOQ for four analytes and of 500 ng/mL for midazolam (IS) were selected to investigate the crosstalk interference among five MRM channels. Similar to carry-over assessment, the cutoff index for crosstalk was also 20%, the ratios of the area in other null channels to that of a LLOQ sample.

#### 2.5.7 Dilution integrity

Dilution integrity was investigated to ensure that samples could be diluted with blank plasma without affecting the real concentration. PZA, EMB, PTO and CFZ samples prepared at different multiple times of HQC were diluted with pooled dog plasma at dilution factors of 2, 5, 10, 20, 50 and 100 in five replicates. The acceptance criteria were consistent with the cutoff indexes of precision and accuracy evaluation (i.e., R.S.D for precision ≤15% and deviation for accuracy within ±15%).

#### 2.5.8 Stability

Stability investigation were conducted to evaluate the stability of four drugs and IS in stock solution, plasma and processed samples under different conditions. The validation comprised short-term temperature stability test, long-term stability test, auto-sampler stability test, and freeze-thaw cycles stability test to cover all factors during sampling and analyzing. All items were assessed at two QC levels (LQC and HQC) in triplicate and shared the same limit as accuracy evaluation. In consideration of practical situation, the low limits of hold time and other conditions for stability tests were described as followed: 24 h for autosampler, room temperature (18 °C), processed samples; 4 h for bench-top, low temperature (4 °C), plasma samples; 1 month for long term, frozen temperature (stock solution, −20 °C; plasma samples, −80 °C); 3 freeze-thaw cycles for freeze-thaw, 12 h for interval time, frozen temperature (−80 °C), plasma samples.

### 2.6 Application of the LC–MS/MS method for pharmacokinetic studies

#### 2.6.1 Animals

All the animal experiments followed the Guidelines for the Care and Use of Laboratory Animals. Beagle dogs (10 ± 0.5 kg) were obtained from Shanghai *Xingang* Experimental Animal Base. The dogs were acclimated in the base for 1 week prior to the experiments, housed under the room temperature with a 12 h light/dark cycle, supplied with standard diet and water. All the dogs were fasted for 12 h before oral administration with free access to water.

#### 2.6.2 Drug administration and plasma sample collection

The dosage selected for dogs was based on the previous studies [11,12] and clinical routine dose, and the standard dose was set as following described: PZA was 93.2 mg/kg; EMB was 20.7 mg/kg; PTO was 15.5 mg/kg; CFZ was 5.2 mg/kg. The dosage conversion involved here between different species based on body surface area.

In the pharmacokinetic study, 18 dogs were randomly divided into three groups and given the combination orally with different dosages (half, standard and double, n = 6 per group). Oral administration was the only dose route to mimic the clinical delivery way. Blood samples were collected via foreleg vein into heparinized tubes prior to the dose and at 0.25, 0.5, 0.75, 1, 1.5, 2, 3, 4, 5, 6, 8, 10, 12, 24, 36, 48 h to describe intact time courses of each individual drug. Then, each blood sample was immediately centrifuged at approximately 3500 *g* for 10 min at 4 °C and the plasma was separated and stored at −80 °C until analysis.

#### 2.6.3 Pharmacokinetic study and data analysis

The pharmacokinetic parameters, including maximum plasma concentration (C_max_), time to maximum concentration (T_max_), area under plasma concentration-time curve from 0 to 24/48 h (AUC_0-24/48h_), area under plasma concentration-time curve from 0 to infinity (AUC_0-∞_) and terminal elimination half-life (t_1/2_), were calculated for each dog using DAS 3.2.8 pharmacokinetic program (Chinese Pharmacology Society) with non-compartmental model. Results are presented as the mean ± S.D.

## 3 Results and discussion

### 3.1 Optimization of LC–MS/MS conditions

**Fig. 1:**
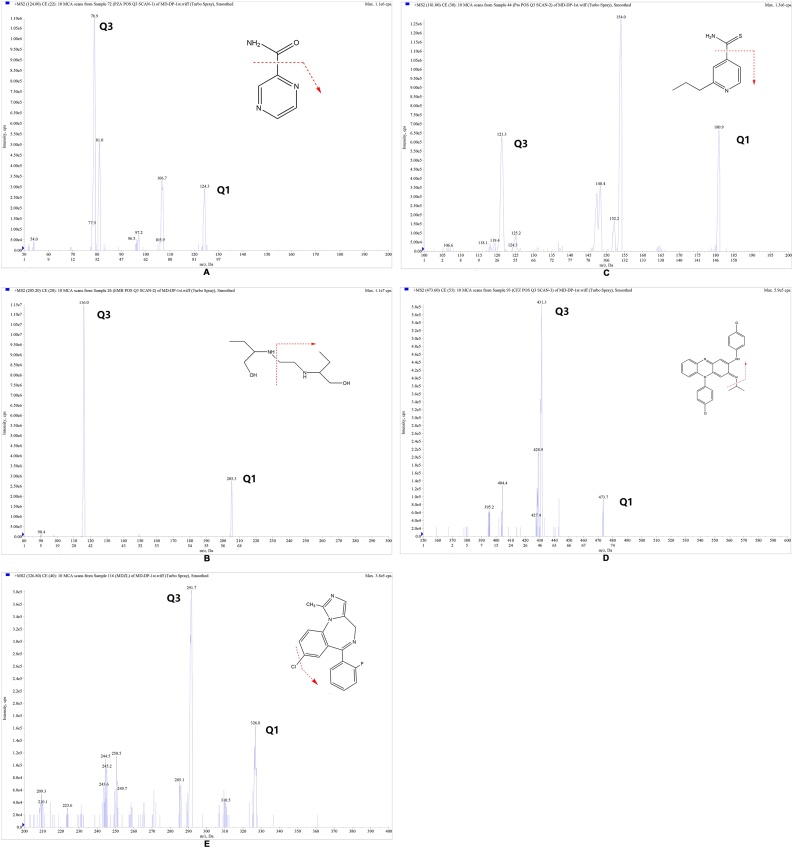
Product ion spectra and chemical structures of pyrazinamide (PZA, A), ethambutol (EMB, B), protionamide (PTO, C), clofazimine (CFZ, D) and midazolam (IS, E), obtained by collision-induced dissociation (CID) of the indicated precursor ions [M+H]^+^. The fragmentation patterns of all analytes and IS are indicated by an arrow on the chemical structure of each analyte. The residual precursor and the product ions, quantifier and qualifier, are shown in the figure. The results are presented as the mass response versus mass-to-charge (*m*/*z*).

To obtain the favorable selectivity and sensitivity for all analytes, several chromatographic and mass spectrometric conditions were optimized. The selection of ionization mode was based on the obtained sensitivity with ESI and atmospheric pressure chemical ionization (APCI) source. The results showed that ESI operated in positive mode provided increased intensity for the analytes compared to APCI. The fragmentations of analytes and IS were manual optimized via precursor ion search of approximately 500 ng/mL of working solution for each compound. The most abundant precursor → product ions in terms of better sensitivity for PZA, EMB, PTO, CFZ and midazolam were found to be *m/z* 205.1 → 116.1, 124.1 → 79.0, 181.1 → 121.1, 473.1 → 431.1 and 326.1 → 291.1, respectively. The product-ion scan spectrum and the fragmentations of the compounds were depicted in Fig. 1. The compound dependent parameters such as DP, EP, CE, CXP were also optimized to obtain the highest signal intensity for all the analytes and IS (Table 1).

Due to the huge discrepancy of polarity and molecular weight among these five compounds (four drugs and one IS), it was difficult to select a suitable chromatographic column to realize good resolution, symmetric peak shapes and appropriate retention times simultaneously. EMB possessing two hydroxyl groups and simple framework behaves great hydrophilia and hardly remains on a general C18 or C8 column. Oppositely, CFZ shows extremely high hydrophobicity because of aromatic structure, which would be strongly retained on lipophilic stationary phase. As for PZA and PTO, the molecular structures are also of high hydrophobicity. To develop a suitable LC method, plenty of the chromatographic conditions, including the mobile phase composition, chromatographic modifiers and different analytical columns were evaluated and optimized to achieve acceptable resolutions and symmetrical peak shapes of the analytes, as well as a short run time. Different solvent systems such as ACN and MeOH with various buffers like ammonium acetate and ammonium formate in different pH and different flow rates were tried meticulously. The several columns at different length or inner diameters, such as Inertsil ODS3 C18 column [25], Ailgent ZORBAX SB-C18 column, Atlantis dC18 column [26], Phenomenex Luna RP-C18 column [27], were applied to this assay for the suitable retention time and separation. Finally, an Agilent SB-Aq column was selected to conduct this assay coming to terms with a suitable chromatographic behavior. The SB-Aq column belonged to C18 column overall, and the routine application and daily maintain were much easier without extra operation burden. A column with 4.6 mm I.D. was the superior and more robust option as the column pressure would be more susceptible to matrix accumulation in a smaller I.D. leading to the variation in chromatographic behavior after hundreds of biological samples. And without proper ammonium acetate or acid in mobile phase, it was observed that no signal occurred in the channel of EMB and PTO and the flickering endogenous substance in plasma interfere the determination of PZA. Thus, 5 mmol ammonium acetate and 0.2% formic acid were added into water. The pH of the mobile phase was less than 3 which could be covered well by the SB-Aq column as it was capable of wide working pH range. Methanol was the only candidate for organic phase as the poor solubility of EMB in acetonitrile. The special gradient during 0.5 min and 1.5 min was designed to achieve maximum separation between PZA and the flickering interference. Further, the reproducibility (%CV) in the measurement of retention time for the analytes was less than 0.5% for 100 injections. This LC method has been successfully transferred to another LC–MS/MS system (Shimazdu, LC–MS 8040) equipped with LC-20A with similar chromatography performance.

### 3.2 Selection of internal standard

These four drugs possess a huge extent of polarity and solubility and present a big dispersion in retention times, so it is difficult to select a single suitable internal standard in the LC–MS/MS condition described above. Several compounds were tested strictly. The phenacetin containing a benzene ring have a similar behavior with PTO on the column but the matrix effect is a unsatisfied with only about 60% which would result in the decrease of peak area in a big batch. Another compound, carbamazepine, has also been studied in consideration of the poly-cyclic structure and strong signal in mass spectrum. But when it come to a long batch, the peak height and area dropped after dozens of plasma samples continuously. This signal attenuation might be caused by the accumulation of the matrix in the column, resulting in more matrix being co-eluted in latter sample than the former. At last, the midazolam with a proper retention time and symmetrical peak shape was chosen as the internal standard, no worrying about the signal attenuation. The method validation proved the fitness of midazolam in this LC–MS/MS method.

### 3.3 Sample preparation

Aiming to establish a high throughput and robust method to determinate four drugs simultaneously, the sample preparation is the limiting factor. Protein precipitation (PPT) and liquid-liquid/solid-phase extraction (LLE/SPE) are routine sample pretreatment strategies. In this study, methanol was the only option for PPT for EMB have poor solubility in acetonitrile, which would cause the accuracy loss. For another, PTO and CFZ cannot be solved in ethyl acetate or *tert*-Butyl methyl ether (TBME) which are the common solvent for liquid-liquid extraction. Thus, the pretreatment of liquid-liquid extraction presents a depressing result that these four components cannot be totally extracted simultaneously at one step and it would cause a large burden for pretreatment. For PPT, different ratios of precipitant-to-plasma (v/v) were also tested. However, no significant difference on matrix effect or recovery at three QC concentrations was found among the ratios of 3, 5 and 7. At last, 3:1 (v/v), 150 μL methanol for 50 μL plasma, was chosen for the PPT ratio. Although convenient and simple, PPT is weak in removing salt and endogenous substances absolutely, and these remaining endogenous substances would be co-eluted with the target compounds and influence the effect of ionization which had been observed during method development. So, dilution strategy was applied to reduce matrix effect and methanol: water: formic acid (20:80:0.02, v/v/v) was chosen as the diluent to ensure a symmetry peak shape and good solubility of the compounds in the mixture.

### 3.4 Method validation

#### 3.4.1 Selectivity

**Fig. 2:**
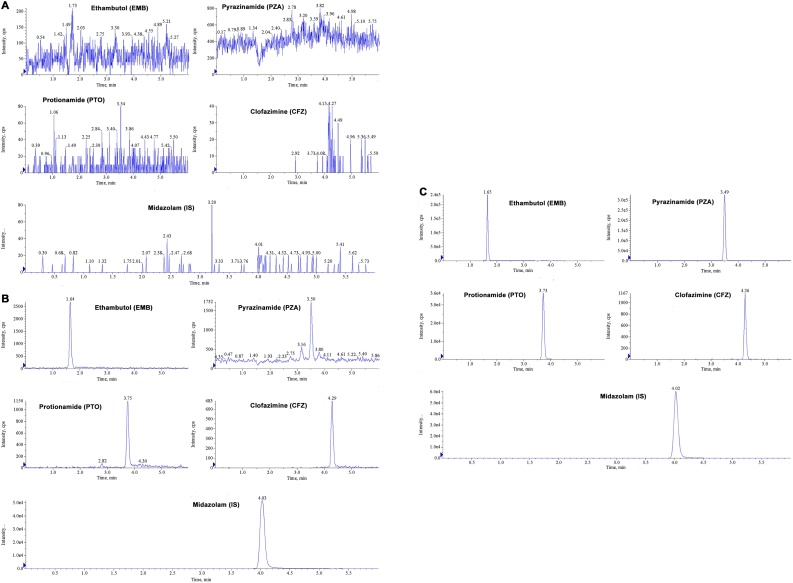
Extraction ion chromatograms (XIC) of four analytes and IS, respectively. Beagle dog blank plasma (A), blank dog plasma spiked with LLOQ of four analytes and IS (B) and plasma from a subject 1.5 h post dose of the combination drugs (C).

In order to insure the undisturbed peaks, endogenous and exogenous tests were designed for selectivity. For exogenous tests, no irrelevant signal was spotted on the spectrum under the LC and MS conditions in this assay, such as ammonium acetate buffer, large gradient et al. For endogenous tests, six blank plasma samples were derived from six different individuals with the same age as the dogs for pharmacokinetics studies. The chromatograms of each selectivity sample were compared with LLOQ samples and real samples for further assessment in real situations. The retention times of PZA, EMB, PTO, CFZ and midazolam were at 3.5 min, 1.6 min, 3.8 min, 4.3 min and 4.0 min, respectively. All compounds were proved to be safe from endogenous interference in each channel. Fig. 2 shows the typical chromatograms of a blank plasma sample, LLOQ sample, and real sample obtained at 1.5 h after the oral administration. The baselines in five channels were steady on the whole except for PZA. Due to the low molecule weight of PZA (MW = 123), the basic intensity of mass spectrum in PZA channel was around 500cps. A small notch was observed at around 1.5 min in baseline (Fig. 2.A), which should be due to the co-elution matrix from column that inhibited the response. This signal suppression indicated peaks of EMB should be suppressed, described as matrix effect, at its retention time. The full evaluation of matrix effect has been presented as following statement and it has been verified that the matrix effect was constant across the calibration ranges and had no impact on the accuracy of quantitation.

#### 3.4.2 Calibration curve and lower limit of quantification

The hybrid calibration curve containing eight different concentration points for each drug proved to have good curve fitting characteristics with a 1/x^2^ weighing coefficient. The calibration equation for PZA was y = 0.00383x − 0.00539 (20–5000 ng/mL, r^2^ = 0.9990); for EMB was y = 0.0611x + 0.0307 (1–500 ng/mL, r^2^ = 0.9990); for PTO was y = 0.0614x - 0.0758 (1–500 ng/mL, r^2^ = 0.9996); for CFZ was y = 0.00965x − 0.00967 (1–200 ng/mL, r^2^ = 0.9990). The LLOQs in dog serum were set at 20 ng/mL for PZA and 1 ng/mL for EMB, PTO and CFZ and the S/N ratios were much greater than 10 (Fig. 2.B) and met the needs of Beagle pharmacokinetics studies. If necessary, the LLOQs of this method could be lower and regulated as needed.

#### 3.4.3 Accuracy and precision

Eighteen replicate samples at each QC concentrations were analyzed in three separate runs, six for each run. Accuracy was evaluated by the ratios of the calculated concentrations to the nominal values and the precision was presented as R.S.D. of the results. Table 2 shows the summary of the data obtained in three separate QC runs. QC with four levels covering the quantitation span served as proper samples for test and similar accuracy and precision of QC in four concentration levels were observed, which was an indirect evidence for the good linearity of calibration curves. These results verified the accuracy and reliability of the LC–MS/MS method, also the PPT strategy, for the determination of the four anti-TB drugs in dog plasma.

**Table 2:** Results of precision and accuracy of PZA, EMB, PTO and CFZ in Beagle dog plasma (n = 6).

	Concentration	Intra-day		Inter-day	
		Accuracy (%)	RSD (%)	Accuracy (%)	RSD (%)
PZA	20	96.0	3.9	98.5	11.8
	60	98.3	3.5	99.7	4.8
	450	102.2	4.5	102.2	7.2
	4000	93.9	2.6	95.5	5.9
EMB	1	99.1	5.0	99.6	9.4
	3	101.8	3.1	103.4	4.0
	40	107.3	3.2	98.8	7.2
	400	97.0	2.8	94.3	3.6
PTO	1	100.5	1.7	103.6	1.7
	3	96.0	3.1	95.0	3.1
	40	100.8	2.3	94.5	2.3
	400	96.2	1.8	96.3	1.8
CFZ	1	94.2	9.3	105.4	13.0
	3	99.3	7.5	95.9	6.7
	15	96.4	3.6	93.5	7.0
	160	98.6	3.2	95.5	5.6

#### 3.4.4 Matrix effect and recovery

The matrix effect was estimated by area ratios between standard samples and matrix samples at three concentrations (low, medium and high). The results demonstrated that the ion suppression had little effect on ionization for PZA, CFZ and IS own to the appropriate sample pretreatment and gradient elution. For PTO and EMB, although the serve ion suppression existed in ion source during the ionization, the matrix effect showed no big difference across three concentrations (low, medium and high), proved to be consistent, precise and reproducible and would not disturb the accuracy of quantitation. And during the method development, no attenuation in signal intensity was observed for each compound in a big batch (more than 100 samples). It could be inferred that each sample was analyzed at the same level of matrix effect across the batch. This great consistency testified the robust of the method.

The recovery, indicating the extraction efficiency of compounds from biological substrate, was evaluated at three concentrations. The recoveries for all QC samples were within the range of 92.7%–106.8% across the concentrations. Therefore, it had been proved that this extraction procedure was accurate and reproducible within the scope of standard curve.

Table 3 summarizes the results of recovery (extraction efficiency) and matrix effect. The data was shown in form of mean and S.D.

**Table 3:** Results of recovery (extraction efficiency) and matrix effect of PZA, EMB, PTO, CFZ and IS in Beagle dog plasma (n = 6).

	PZA			EMB			PTO			CFZ			IS		
	LQC	MQC	HQC	LQC	MQC	HQC	LQC	MQC	HQC	LQC	MQC	LQC	MQC	LQC	MQC
Nominal concentration (ng/mL)	60	450	4000	3	40	400	3	40	400	3	15	160	50	50	50
Recovery% (R.S.D.%)	92.7(1.2)	93.6(4.9)	94.6(2.8)	103.2(1.9)	99.4(1.9)	99.9(2.6)	96.7(0.8)	94.0(1.1)	101.2(1.4)	102.3(4.4)	101.9(1.9)	101.6(2.7)	100.3(2.4)	106.8(1.5)	106.1(1.0)
Matrix effect% (R.S.D.%)	90.1(1.0)	92.7(1.3)	88.8(2.1)	64.1(4.4)	66.1(2.3)	69.6(0.6)	73.6(1.0)	68.2(3.2)	60.0(0.5)	89.7(2.4)	85.5(0.3)	88.4(1.4)	91.0(1.3)	91.3(2.2)	93.4(1.1)

#### 3.4.5 Hemolysis evaluation

**Fig. 3:**
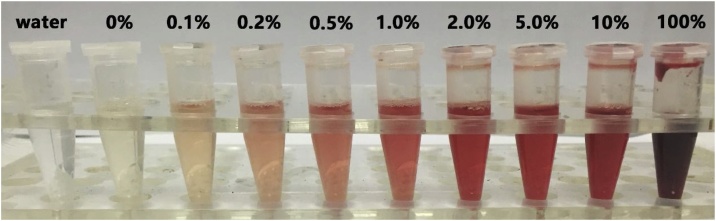
The hemolysis samples, left to right-water, no-hemolytic plasma, 0.1%, 0.2%, 0.5%, 1%, 2%, 5%, 10% and 100%.

**Fig. 4:**
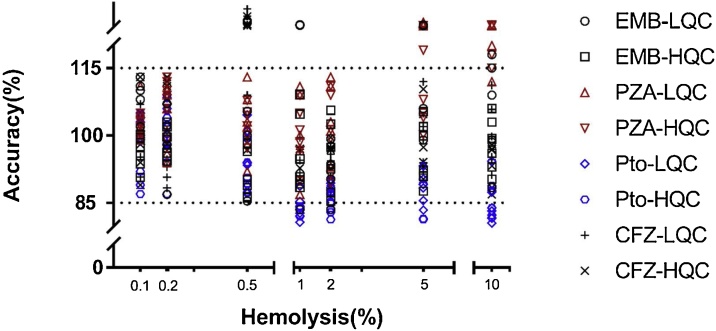
The accuracy of QC samples with different degrees of hemolysis. Two dotted lines are the upper and lower bound of the acceptance criteria.

Hemolysis evaluation focused on the accuracy of quantitation with different hemolytic extent. The results showed that when the hemolytic degrees exceeded 0.5%, the accuracy could not be guaranteed for each sample and the extremely abnormal responses were also observed in the hemolytic matrix effect test. And, more remarkable, the results in this hemolysis evaluation assay was much more rigorous and the actual degrees of hemolysis could be greater than what we attempted to conduct because this simple and homemade method for making hemolytic plasma was of relative quantitation for degrees of hemolysis and standard zero-hemolysis plasma probably was not pure, may already with little hemolysis to some extent. Nonetheless, these outcomes surely demonstrated that the hemolysis beyond 0.5% might influence the determination of the plasma samples and should be avoid when sampling. The prepared hemolysis plasma samples and the accuracy of QC samples with different degrees are shown in Fig. 3, Fig. 4, respectively.

#### 3.4.6 Stability

The stability was studied under various conditions mainly covering the whole process. The mean values and S.D. of the ratios between the measured and theoretical concentrations were used for stability evaluation. The results were shown in Table 4, and no significant drug content decays were observed within the scope of target conditions and indicated that PZA, EMB, PTO and CFZ had acceptable stabilities to guarantee the accuracy under the test conditions with the accuracy 86.6–113.2%.

**Table 4:** Results of stability of PZA, EMB, PTO and CFZ in Beagle dog plasma (n = 3).

	%Stability recoveries(Mean ± SD)							
	Freeze-thaw (−80 °C)		Long-term 40 days (−80 °C)		Auto-sampler 27 h (18 °C)		Bench-top 4 h (4 °C)	
	LQC	HQC	LQC	HQC	LQC	HQC	LQC	HQC
PZA	106.3 ± 3.0	98.6 ± 0.7	97.9 ± 2.0	88.6 ± 0.9	101.1 ± 1.6	102.5 ± 3.3	95.2 ± 3.4	95.4 ± 2.5
EMB	106.6 ± 6.2	98.0 ± 2.6	109.3 ± 4.7	101.8 ± 0.9	103.2 ± 3.6	94.0 ± 5.0	100.5 ± 4.8	86.6 ± 0.9
PTO	93.5 ± 2.5	101.6 ± 3.7	94.3 ± 2.2	92.5 ± 0.8	102.0 ± 4.3	103.5 ± 2.3	96.9 ± 4.7	96.2 ± 0.9
CFZ	113.2 ± 3.8	102.8 ± 2.3	91.3 ± 1.0	98.5 ± 2.3	95.3 ± 8.6	103.3 ± 1.3	92.5 ± 4.6	96.1 ± 2.0

Results of stability of PZA, EMB, PTO and CFZ in Beagle dog plasma (n = 3).

#### 3.4.7 Dilution integrity, carry-over and crosstalk

The dilution integrity for all analytes with the dilution factors of 2, 5, 10, 20, 50 and 100 was verified by post-diluted accuracy and precision and proved to be within acceptable threshold, below 15% for precision (R.S.D.) and within 85.0–115.0% for accuracy (n = 5). The results showed the reliability of dilution in synchronism or in individual and the samples beyond calibration curve could be determined accurately.

No carry-over and no baseline rise were observed in each channel of the blank sample after ULOQ. No crosstalk appeared among five MRM channels and indicated that the AB SCIEX API-4000 system can serve well as the quantitative tool theoretically.

### 3.5 Application to pharmacokinetic study

**Fig. 5:**
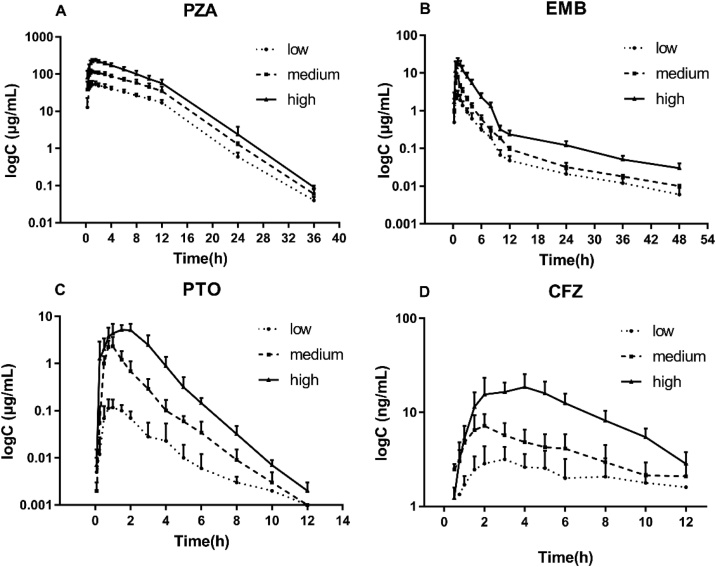
Mean serum concentration versus time profiles of PZA (A), EMB (B), PTO (C) and CFZ (D) following three different oral doses in combination to Beagle dogs (n = 6).

**Fig. 6:**
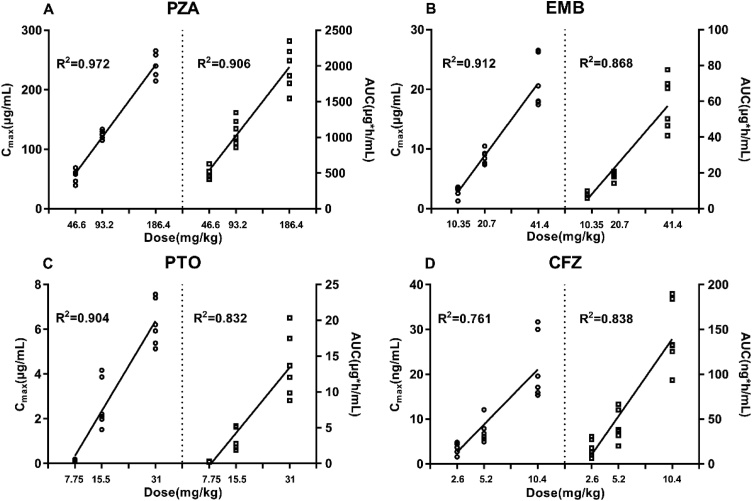
Plasma exposure (C_max_ and AUC) vs dose correlation. Linearity of PZA (A), EMB (B), PTO (C) and CFZ (D) across three different doses following oral administration in combination (n = 6).

Owe to the preliminary study, a four-drug anti-TB combination (PZA, EMB, PTO and CFZ) was optimized systematically and proposed. In this assay, a high throughout and robust LC–MS/MS method was established and had been successfully applied in Beagle dogs to study the properties of pharmacokinetics for each drug in combination at three stepped dosages. Dogs were orally administrated with the combination according to the protocol. The routine dosages for each drug in clinical use should have been covered roughly by this dose-ranging study. Mean plasma concentration-time curves of different groups are show in Fig. 5. The data analyzed by non-compartmental method are shown in Table 5. C_max_ and AUC_0-τ_ showed dose-dependent increase and possessed favorable linear relationship (R^2^ ≧ 0.76) across three stepped dosages for each drug in Fig. 6.

**Table 5:** Pharmacokinetic parameters of combination drugs in three different dosages.

Compounds	Parameter	Unit	Low Dose	Medium Dose	High Dose
PZA	T_max_	h	1.13 ± 0.54	1.04 ± 0.60	1.33 ± 0.26
	T_1/2_	h	2.82 ± 0.08	2.70 ± 0.21	2.62 ± 0.10
	C_max_	μg/mL	57.0 ± 11.9	125 ± 6.70	241 ± 19.2
	AUC_0-τ_	μg h/mL	50.2 ± 7.60	1080 ± 187	1970 ± 298
	AUC_0-∞_	μg h/mL	50.2 ± 7.60	1080 ± 188	1970 ± 298
EMB	T_max_	h	1.25 ± 0.94	0.83 ± 0.20	0.92 ± 0.38
	T_1/2_	h	11.77 ± 1.31	12.66 ± 1.88	11.97 ± 0.62
	C_max_	μg/mL	2.93 ± 0.89	8.72 ± 1.15	21.1 ± 4.23
	AUC_0-τ_	μg h/mL	7.88 ± 1.22	18.5 ± 2.35	58.7 ± 14.8
	AUC_0-∞_	μg h/mL	7.98 ± 1.23	18.7 ± 2.34	59.2 ± 15.0
PTO	T_max_	h	1.50 ± 1.25	0.83 ± 0.13	1.46 ± 0.51
	T_1/2_	h	1.07 ± 0.29	1.39 ± 0.41	0.97 ± 0.09
	C_max_	μg/mL	0.147 ± 0.043	2.64 ± 1.10	6.27 ± 1.02
	AUC_0-τ_	μg h/mL	0.267 ± 0.041	3.33 ± 1.46	13.7 ± 4.47
	AUC_0-∞_	μg h/mL	0.269 ± 0.041	3.34 ± 1.46	13.7 ± 4.47
CFZ	T_max_	h	3.17 ± 0.98	1.92 ± 0.20	2.75 ± 1.08
	T_1/2_	h	3.41 ± 1.31	4.03 ± 1.16	3.63 ± 1.49
	C_max_	ng/mL	3.69 ± 1.32	7.24 ± 2.58	21.6 ± 7.32
	AUC_0-τ_	ng h/mL	17.5 ± 9.67	42.2 ± 17.7	143 ± 37.1
	AUC_0-∞_	ng h/mL	23.8 ± 11.8	53.1 ± 22.1	147 ± 37.4

## 4 Conclusions

In this study, a specialized LC–MS/MS method for simultaneous determination of the promising anti-TB combination (PZA, EMB, PTO and CFZ) was developed and validated. Compared with published analytical methods (LC–MS/MS or LC-UV), this method shows the advantages of more sensitive with shorter analytical time and smaller sample volume. The improved sensitivity will be helpful for the extension of the studies to adult and children where blood volume for bioanalytical samples may be limited. Moreover, it is the first time that the hemolytic effect of LC–MS/MS methods has been evaluated for anti-TB drugs and the accuracy cannot be guaranteed when the hemolytic degrees exceeded 0.5%. This method was successfully applied to the pharmacokinetics study in Beagle dogs and the results we report here should provide basic information for anti-TB drug development.

## Conflicts of interest

The multi-drug therapy for tuberculosis treatment described here has been patented (International Patent Application Serial No. PCT/US2015/058892)
